# Estimated Substitution of Tea or Coffee for Sugar-Sweetened Beverages Was Associated with Lower Type 2 Diabetes Incidence in Case–Cohort Analysis across 8 European Countries in the EPIC-InterAct Study

**DOI:** 10.1093/jn/nxz156

**Published:** 2019-08-08

**Authors:** Fumiaki Imamura, Matthias B Schulze, Stephen J Sharp, Marcela Guevara, Dora Romaguera, Benedetta Bendinelli, Elena Salamanca-Fernández, Eva Ardanaz, Larraitz Arriola, Dagfinn Aune, Heiner Boeing, Courtney Dow, Guy Fagherazzi, Paul W Franks, Heinz Freisling, Paula Jakszyn, Rudolf Kaaks, Kay-Tee Khaw, Tilman Kühn, Francesca R Mancini, Giovanna Masala, Maria-Dolores Chirlaque, Peter M Nilsson, Kim Overvad, Valeria M Pala, Salvatore Panico, Aurora Perez-Cornago, Jose R Quirós, Fulvio Ricceri, Miguel Rodríguez-Barranco, Olov Rolandsson, Ivonne Sluijs, Magdalena Stepien, Annemieke M W Spijkerman, Anne Tjønneland, Tammy Y N Tong, Rosario Tumino, Linda E T Vissers, Heather A Ward, Claudia Langenberg, Elio Riboli, Nita G Forouhi, Nick J Wareham

**Affiliations:** 1 Medical Research Council Epidemiology Unit, University of Cambridge, Cambridge, United Kingdom; 2 Department of Molecular Epidemiology, Germen Institute of Human Nutrition, Potsdam, Germany; 3 IDISNA Navarra Health Research Institute, Pamplona, Spain; 4 CIBER Epidemiology and Public Health, Madrid, Spain; 5 Department of Epidemiology and Biostatistics, School of Public Health, Imperial College London, London, United Kingdom; 6 Health Research Institute of Balearic Islands (IdISBa), Palma de Mallorca, Spain; 7 CIBER Physiopathology of Obesity and Nutrition, Madrid, Spain; 8 Cancer Risk Factors and Lifestyle Epidemiology Unit, Institute for Cancer Research, Prevention and Clinical Network (ISPRO), Florence, Italy; 9 Andalusian School of Public Health, Institute of Investigation Instituto de Investigación Biosanitaria de Granada (ibs.GRANADA), University of Granada, Granada, Spain; 10 Public Health Division of Gipuzkoa, Instituto BIO-Donostia, Basque Government, CIBERESP, Gipuzkoa, Spain; 11 Department of Nutrition, Bjørknes University College, Oslo, Norway; 12 Department of Endocrinology, Morbid Obesity and Preventive Medicine, Oslo University Hospital Ullevål, Oslo, Norway; 13 Gustave Roussy Institute, Villejuif, France; 14 University Paris–South, Faculty of Medicine, University Versailles–St Quentin, University Paris-Saclay, Villejuif, France; 15 National Institute for Health and Medical Research (INSERM), Center for Research in Epidemiology and Population Health, Villejuif, France; 16 Department of Public Health and Clinical Medicine, Family Medicine, Umeå University, Umeå, Sweden; 17 Department of Clinical Sciences, Lund University, Skane University Hospital, Malmo, Sweden; 18 Section of Nutrition and Metabolism, International Agency for Research on Cancer (IARC–WHO), Lyon, France; 19 Catalan Institute of Oncology, Barcelona, Spain; 20 FCS Blanquerna, Universitat Ramon Llull, Barcelona, Spain; 21 Division of Cancer Epidemiology, German Cancer Research Center, Heidelberg, Germany; 22 Department of Public Health and Primary Care, University of Cambridge School of Clinical Medicine, Cambridge, United Kingdom; 23 Department of Epidemiology, Regional Health Council, IMIB-Arrixaca, Murcia, Spain; 24 Department of Health and Social Sciences, Universidad de Murcia, Murcia, Spain; 25 Department of Cardiology, Aalborg University Hospital, Aarhus, Denmark; 26 Section for Epidemiology, Department of Public Health, Aarhus University, Aarhus, Denmark; 27 Epidemiology and Prevention Unit, Fondazione IRCCS Istituto Nazionale dei Tumori, Milan, Italy; 28 Dipartimento di Medicina Clinica e Chirurgia, Federico II University, Naples, Italy; 29 Cancer Epidemiology Unit, Nuffield Department of Population Health, University of Oxford, Oxford, United Kingdom; 30 Public Health Directorate, Asturias, Spain; 31 Department of Clinical and Biological Sciences, University of Turin, Turin, Italy; 32 Unit of Epidemiology, Regional Health Service ASL TO3, Grugliasco, Italy; 33 Julius Center for Health Sciences and Primary Care, Cardiovascular Epidemiology, University Medical Center Utrecht, Utrecht, Netherlands; 34 National Institute for Public Health and the Environment, Bilthoven, Netherlands; 35 Danish Cancer Society Research Center, Copenhagen, Denmark; 36 Cancer Registry and Histopathology Department, “Civic-M.P. Arezzo” Hospital, Ragusa, Italy

**Keywords:** diabetes, epidemiology, dietary guidelines, beverages, sugar-sweetened beverages

## Abstract

**Introduction:**

Beverage consumption is a modifiable risk factor for type 2 diabetes (T2D), but there is insufficient evidence to inform the suitability of substituting 1 type of beverage for another.

**Objective:**

The aim of this study was to estimate the risk of T2D when consumption of sugar-sweetened beverages (SSBs) was replaced with consumption of fruit juice, milk, coffee, or tea.

**Methods:**

In the European Prospective Investigation into Cancer and Nutrition (EPIC)–InterAct case–cohort study of 8 European countries (*n* = 27,662, with 12,333 cases of incident T2D, 1992–2007), beverage consumption was estimated at baseline by dietary questionnaires. Using Prentice-weighted Cox regression adjusting for other beverages and potential confounders, we estimated associations of substituting 1 type of beverage for another on incident T2D.

**Results:**

Mean ± SD of estimated consumption of SSB was 55 ± 105 g/d. Means ± SDs for the other beverages were as follows: fruit juice, 59 ± 101 g/d; milk, 209 ± 203 g/d; coffee, 381 ± 372 g/d; and tea, 152 ± 282 g/d. Substituting coffee for SSBs by 250 g/d was associated with a 21% lower incidence of T2D (95% CI: 12%, 29%). The rate difference was −12.0 (95% CI: −20.0, −5.0) per 10,000 person-years among adults consuming SSBs ≥250 g/d (absolute rate = 48.3/10,000). Substituting tea for SSBs was estimated to lower T2D incidence by 22% (95% CI: 15%, 28%) or −11.0 (95% CI: −20.0, −2.6) per 10,000 person-years, whereas substituting fruit juice or milk was estimated not to alter T2D risk significantly.

**Conclusions:**

These findings indicate a potential benefit of substituting coffee or tea for SSBs for the primary prevention of T2D and may help formulate public health recommendations on beverage consumption in different populations.

## Introduction

Beverage consumption is one of the modifiable risk factors for noncommunicable diseases, including type 2 diabetes (T2D) (1–4). Observational studies have indicated a benefit or harm of different types of beverages for the primary prevention of noncommunicable diseases. For instance, adults consuming ≥1 daily serving of sugar-sweetened beverages (SSBs) are likely to experience a 13% greater incidence of T2D (5), whereas adults consuming 2–3 daily servings of coffee or tea are likely to experience a 15–25% lower incidence of T2D (6–8). This evidence has been supported by analyses that evaluated associations of different types of beverages with incident T2D separately. Evidence remains limited, however, on the potential benefit of substituting 1 type of beverage for another by examining different types of beverages simultaneously (9).

Effects of substituting alternative beverages for SSBs are of particular interest to inform clinical and public health recommendations. This is because consumption of SSBs has been related to cardiometabolic burden worldwide (2–4) and because multiple alternatives of beverages to SSBs are available, with diversity in sales and consumption across countries (10, 11). The effects on cardiometabolic risks have been tested in trials that, for example, examined the effect of replacing SSBs with milk, fruit juice, water, or noncaloric artificially-sweetened beverages (ASBs) on cardiometabolic risk factors (12–14). Clinical endpoints have been studied only in a few cohorts for stroke (15) or T2D (9, 16–18) in the United States and the United Kingdom (15–17). We aimed to provide further evidence by evaluating participants in the European Prospective Investigation into Cancer and Nutrition (EPIC)–InterAct consortium (19), a case–cohort study of T2D that reported associations of selected beverages with T2D, including tea (20), milk (21), fruit juice (22), ASBs (22), and SSBs (22), but had not yet evaluated coffee, milk beverages, or water. Accounting for the available evidence (4–8, 19–24), we hypothesized that substituting tea or coffee for SSBs could lower risk of T2D, whereas substituting the other beverages for SSBs would not affect the risk.

## Methods

### Study population

EPIC-InterAct is a prospective case–cohort study nested within the EPIC study cohort from 8 European countries (France, Italy, Spain, the United Kingdom, the Netherlands, Germany, Sweden, and Denmark) (19). From a total of 340,234 cohort participants (3.99 million person-years of follow-up) with stored blood and reported diabetes status, EPIC-InterAct identified 16,835 adults randomly selected (subcohort) and ascertained 12,403 incident cases of T2D occurring by 31 December, 2007; the identified cases included 778 cases in the subcohort (19, 25). In the current study, we excluded adults with prevalent diabetes (*n* = 548) or without information on T2D (*n* = 129) or baseline diet (*n* = 117). After these exclusions, 27,662 adults were evaluated in our analysis (*n* cases = 12,333) (**Supplemental Figure 1**). All participants gave written informed consent. The study was approved by local ethics committees and the Internal Review Board of the International Agency of Research on Cancer.

### Ascertainment of T2D

A diagnosis of incident T2D was defined as self-report of physician's diagnosis verified by at least 1 independent source, including multiple information sources reviewed by each participating center (19): self-report, linkage to primary-care registers, secondary-care registers, medication use (drug registers), hospital admissions, and mortality data (**Supplemental Table 1**). Information from any follow-up visit or external evidence with a date later than the baseline visit was used. In Denmark and Sweden, incident cases were identified via local and national diabetes and pharmaceutical registers and were considered to be verified. Follow-up was to the date of diagnosis, 31 December, 2007, or the date of death, whichever occurred first.

### Beverage consumption

In EPIC, dietary consumption was assessed by a dietary FFQ or diet history harmonized across EPIC cohorts (26–29). Five beverages were used as the primary exposures: SSBs (carbonated/soft drinks or diluted syrups, and sweetened milk beverages), fruit juice (fruit or vegetable juices, and fruit concentrates), milk, coffee (caffeinated or decaffeinated), and tea (**Supplemental Table 2** for classification). We included sweetened milk beverages (e.g., milkshakes) as SSBs, not as milk, because of their presumably high sugar contents and their significant positive association with T2D incidence, similar to SSB consumption, in the EPIC-Norfolk study using 7-d food records (18). Based on published reviews and previous EPIC-InterAct analyses, we combined decaffeinated coffee with coffee (8) and vegetable juice with fruit juice (22). Reported consumption of each beverage had moderate correlations—adjusted for age, sex, and energy intake—with corresponding measures of 24-h recalls on average across countries (*n* = 2347): *r* = 0.33 for SSB, *r* = 0.38 for fruit juice, *r* = 0.52 for milk, *r* = 0.60 for coffee, and *r* = 0.52 for tea.

We separately evaluated ASBs (available among 82.8% of participants), decaffeinated coffee (72.1%), vegetable juice (57.0%), and water (58.4%) (Supplemental Table 1). Possible alternative groupings (e.g., classifying sweetened milk beverages as milk rather than SSBs) were evaluated additionally. These beverages were evaluated in the secondary analyses because the missing information (not at random) could limit validity of estimating absolute incidence and because these beverages were assumed to be irregularly consumed and thus the validity of self-reported consumption of these beverages was considered unclear. Alcohol, evaluated previously in EPIC-InterAct (24), was used as a covariate because alcoholic beverages cannot be considered as an alternative to the other beverages in practice.

### Other study variables

At baseline, weight, height, and waist circumference were measured directly in every center, except waist circumference was not assessed in Oxford, UK, and Umea, Sweden (19). Sociodemographic factors, smoking status, physical activity, menopausal status and use of hormone replacement therapy (women only), use of medication, and prevalent diseases were self-reported (19, 30). Food groups were derived from dietary questionnaires or history (28) and considered as dietary confounders. Total energy intake was estimated from European food composition tables and centrally analyzed for EPIC (28). As an indicator of under-, normal, or overreporting of individuals’ diet, we calculated a ratio of total energy intake to total energy requirement as previously performed in EPIC-Spain (31) and categorized the ratio into the 3 levels by 1.0 ± 2 × within-individual SD of the ratio (31).

Serum triglycerides, HDL cholesterol, high-sensitivity C-reactive protein (hsCRP), and hemoglobin A1c (HbA1c) were measured at Stichting Ingenhousz Laboratory using serum samples stored at −196°C (or −150°C in Denmark), with exception of Umeå, where plasma samples were used (details in **Supplemental Methods**). These markers were additionally examined as potential mediators for associations of beverage consumption with incident T2D.

### Statistical analysis

All analyses were performed using Stata version 14.0 software (Stata). Outlying values of beverage consumption were Winsorized (replaced with each mean +3 × SD, country-specific) to reduce their influence. The prospective association between consumption of each type of beverage (SSBs, fruit juice, milk, coffee, and tea) and incident T2D was evaluated using Prentice-weighted Cox regression for every country separately, with age as the underlying timescale, providing estimates of HRs and 95% CIs (32). HR per 250 g/d of each beverage was estimated to evaluate the same quantity of each beverage; sensitivity analyses assigned different amounts to different beverages [e.g., 150 g/d for tea (33)] accounting for varying portion sizes per serving. Country-specific estimates were then pooled across countries by multivariable random-effects meta-analysis (34). Models were adjusted for different types of beverages mutually and for potential confounders: research center, sex, education level, occupation, marital status, menopausal status and use of hormone replacement therapy (only for women), family history of diabetes, prevalent clinical conditions (hypertension, dyslipidemia, cardiovascular diseases, and cancer), and the aforementioned quality indicator of dietary reporting (31). We also adjusted for lifestyle factors: smoking status, physical activity level, dietary supplement use, alcohol consumption, and dietary consumption (total energy intake, vegetables, fruits, nuts, cheese, yogurt, red meats, processed meats, fish, confectionary, and cereals). We further adjusted for BMI (in kg/m^2^) and waist circumference because obesity status may have altered beverage consumption (22). We additionally tested nonlinearity of an association between each of the beverages and incident T2D by meta-regression with restricted cubic spline.

We estimated rate differences (RDs) in T2D incidence comparing different amounts of beverage servings (e.g., between 250 and 0 g/d), using parameter estimates from Cox regression models. RD for each beverage was estimated based on the absolute incidence rate of T2D of adults consuming ≥250 g/d of each beverage. In the main analysis, difference in 10-y rates was modeled using a weighted average of the cumulative incidence of T2D in each country-specific subcohort, weighted for sampling probability (35). Differences during 5 and 20 y of follow-up were also estimated. CIs were estimated using bootstrapping (1000 iterations).

We estimated the potential effects of substituting 250 g/d of 1 type of beverage for the same quantity of another type of beverage (16). The substitution effect was estimated as the difference in effect measures (e.g., β_coffee_ – β_SSB_ for the association of substituting coffee for SSB), as previously conducted (15–17). This modeling produces, for example, an estimated change in T2D incidence if an individual decreased SSB and then increased coffee consumption, whereby the interpretation makes an assumption of causality for 2 exposure variables. As the secondary analysis, we also modeled substitution between 2 beverages by an average amount of each beverage in each country, not assuming serving sizes of beverages.

Sensitivity analyses were performed to examine the impact of various assumptions on the results. We estimated HRs for substitution between 250 g/d of SSBs and 150 g/d of 1 of the other beverages, considering possibly different amounts/serving across beverages; for beverage variables without Winsorization; and for beverages with log-transformation. Multiple imputation (20 data sets) was performed to investigate the impact of missing covariate data (36); the main results were from a single imputation in which between-imputation variability was considered small (0.3% of total variability). We assessed influences of reverse causation by censoring T2D cases diagnosed within the first 6 y (approximately the midpoint of follow-up time in EPIC-InterAct). We also examined the influence of errors in dietary measurements (21, 37). Using dietary estimates from single 24-h recalls in a subset (*n* = 2347) as a reference for dietary questionnaire or history data, we conducted multivariable calibration for HRs for different beverages by computing

(1) $$\begin{equation*}
{{\rm{\beta }}_{{\rm{calibrated}}}} = {{\rm{\beta }}_{{\rm{noncalibrated}}}} \times {\left( {{\sum\nolimits _{{\rm{total}}}}-{\sum\nolimits _{{\rm{error}}}}} \right)^{ - 1}}{\sum\nolimits _{{\rm{total}}}}
\end{equation*}$$

where β is a vector of regression coefficients, log(HRs), and ∑ is the total and error variance–covariance matrices of beverage consumption estimates (37).

We tested if the estimated substitution effects were consistent across country, baseline age, sex, smoking, BMI, and the measure of under- or overreporting of dietary data. Heterogeneity by country was assessed by *I*^2^. For the other variables, we performed stratified analyses followed by meta-regression testing between-strata heterogeneity. We also examined if associations of interest were mediated by triglycerides, HDL cholesterol, hsCRP, and HbA1c by including these variables in the model adjusting for potential confounders.

## Results

Participants’ characteristics at baseline are presented in **Table 1**. Mean ± SD consumption of SSBs was 55 ± 105 g/d in the subcohort. Means ± SDs for the other beverages were as follows: fruit juice, 59 ± 101 g/d; milk, 209 ± 203 g/d; coffee, 381 ± 372 g/d; and tea, 152 ± 282 g/d. Different types of beverage consumption were not correlated or weakly correlated mutually (*r* = −0.02 to −0.23) (data not shown). Socioeconomic and lifestyle factors and prevalent comorbidity showed complex associations with beverage consumption (**Supplemental Table 3**). For example, longer education history was significantly related to higher consumption of fruit juice, coffee, or tea, but lower consumption of SSBs and milk (*P* < 0.007 for each). Higher consumption of fruit juice was related to lower use of dietary supplements and greater prevalence of hypertension, dyslipidemia, and cardiovascular diseases (*P* < 0.001 for each). Underreporting of dietary intakes was more prevalent among consumers of tea (12%) or coffee (10%) than among consumers of other beverages (<10%).

**TABLE 1:** Baseline characteristics of study participants in the EPIC-InterAct case–cohort study^1^

	Subcohort^2^ (*n* = 16,103)	Cases of diabetes (*n* = 12,333)
Age, y	52 ± 9.0	56 ± 7.7
Women, %	62	50
Education ≥high school, %	21	13
Currently employed, %	51	45
Current smokers, %	26	28
Physical activity, ≥moderately active, %	43	37
Postmenopausal,^3^ %	51	34
Hormone therapy,^3^ %	15	14
Family history of diabetes,^4^ %	23	38
Prevalent conditions, %		
Hypertension	19	38
Dyslipidemia	18	36
Cardiovascular disease	3.0	7.0
Dietary supplement use, %	39	39
Dietary consumption		
Energy, MJ/d	9.0 ± 2.8	9.1 ± 3.1
Alcohol,^5^ servings/d	1.3 ± 2.0	1.5 ± 2.5
Fiber, g/d	23 ± 8.0	23 ± 8.4
Vegetables, g/d	184 ± 120	180 ± 126
Fruits, g/d	235 ± 191	229 ± 201
Processed meat, g/d	37 ± 33	42 ± 39
Yogurt, g/d	63 ± 88	59 ± 91
Confectionery, g/d	25 ± 51	29 ± 69
Ratio of energy intake to energy requirement	0.92 ± 0.30	0.90 ± 0.30
Underreporters of dietary consumption, %	11.0	14.0
BMI, kg/m^2^	26 ± 4	30 ± 5
Waist circumference, cm	86 ± 13	97 ± 13
Hemoglobin A1c, mmol/mol	36 ± 4.8	43 ± 10
Triglycerides, mmol/L	1.4 ± 0.9	2.0 ± 1.3
HDL cholesterol, mmol/L	1.5 ± 0.4	1.2 ± 0.4
C-reactive protein, mg/L	2.2 ± 3.9	3.8 ± 5.2

1 For continuous and categorical variables, respectively, means ± SDs and percentages are presented. A case–cohort design was undertaken in which the subcohort and incident cases of type 2 diabetes were independently sampled from the eligible participants in the EPIC cohort. By design, 774 incident cases were also in the subcohort. EPIC, European Prospective Investigation into Cancer and Nutrition.

2 Definitions of each type, percentage of nonconsumers and of those consuming >250 g/d, and characteristics by beverage consumption are presented in Supplemental Tables 2 and 3.

3 Calculated among women.

4 Not available in 2 research centers and thus evaluated among 8850 adults in the subcohort and 6752 cases.

5 Consumption levels in total alcoholic beverages were calculated in each cohort. One serving was defined as 150 mL.

Positive associations with incidence of T2D were seen for SSBs and for milk, whereas inverse or null associations were seen for coffee, tea, and fruit juice (**Table 2**). HRs of SSBs indicated 44% higher T2D incidence (95% CI: 28%, 62%) per 250 g/d increment with adjustment for demographic characteristics only. The association was attenuated but still significant after adjustment for socioeconomic and lifestyle covariates and for adiposity measures. In the most adjusted model, milk consumption was weakly but positively associated with T2D incidence, whereas coffee consumption and tea consumption were inversely associated. Fruit juice was not significantly associated. There was no significant evidence of departure from linearity (**Supplemental Figure 2**).

**TABLE 2:** Prospective associations between habitual consumption of beverages and incidence of type 2 diabetes in 8 countries in the EPIC-InterAct case–cohort study (*n* = 27,662)^1^

	Sugar-sweetened beverages	Fruit juice	Milk	Coffee	Tea
HR (95% CI) per 250 g/d^2^					
Adjusted for demographic variables^3^	1.44 (1.28, 1.62)	0.99 (0.91, 1.07)	1.06 (0.96, 1.17)	0.97 (0.93, 1.01)	0.90 (0.88, 0.93)
+ The other potential confounders	1.25 (1.14, 1.37)	0.98 (0.91, 1.05)	1.10 (1.02, 1.18)	0.92 (0.89, 0.95)	0.90 (0.86, 0.95)
+ BMI, waist circumference	1.18 (1.08, 1.28)	1.06 (0.96, 1.17)	1.10 (1.02, 1.19)	0.91 (0.89, 0.94)	0.93 (0.87, 0.98)
Rate difference (95% CI) comparing 250 g/d with 0 serving/d^4^					
Adjusted for demographic variables^3^	+13 (+8.7, +17)	−3.2 (−7.2, +0.9)	+1.0 (−0.9, +2.9)	−2.0 (−3.5, −0.5)	−4.8 (−7.8, −1.9)
+ The other potential confounders	+8.9 (+2.1, +16)	−0.5 (−6.1, +5.0)	+3.5 (+0.7, +6.2)	−3.3 (−5.8, −0.8)	−3.6 (−6.9, −0.3)
+ BMI, waist circumference	+7.4 (−1.6, +16)	+1.1 (−5.6, +7.8)	+4.3 (+0.8, +7.7)	−4.0 (−7.6, −0.4)	−3.1 (−7.6, +1.5)

1 EPIC, European Prospective Investigation into Cancer and Nutrition.

2 12,333 cases were evaluated. From the InterAct subcohort, 774 cases/192,287 person-years contributed. Five beverages were evaluated simultaneously using country-specific Prentice-weighted Cox proportional hazard regression models. Country-specific estimates were pooled by multivariable random-effects meta-analysis.

3 Five beverages were adjusted mutually for each other. Demographic covariates included recruitment centers, age, and sex. Further adjustment for potential confounders included education, marital status, hormone replacement therapy, menopausal status, history of oral contraceptive use, hypertension, dyslipidemia, family history of diabetes, prevalent diseases (coronary heart disease and stroke), smoking, physical activity, alcohol consumption, dietary supplement use, and dietary consumption (total energy intake, vegetables, fruits, nuts, cheese, yogurt, red meats, processed meats, fish, confectionary, and cereals).

4 Per 1000 persons × 10 y (on average, 39.5/10,000 person-years; 95% CI: 36.8, 42.5).

In sensitivity analyses, the findings for SSBs, coffee, and tea were similar to results from the main analyses, whereas findings for milk and fruit juice were different (**Supplemental Tables 4** and **5**). Milk consumption was not associated with T2D, after excluding under- or overreporters based on estimated energy intake and energy requirement, calibrating for measurement error, or censoring events in the first 6 y. After accounting for measurement error, fruit juice consumption was positively associated with T2D. In analyses assessing whether or not physiological markers mediated an observed association, the findings for milk and tea were attenuated toward the null, potentially mediated by the physiological markers, whereas findings for SSBs and coffee remained significant without marked changes.

**FIGURE 1:**
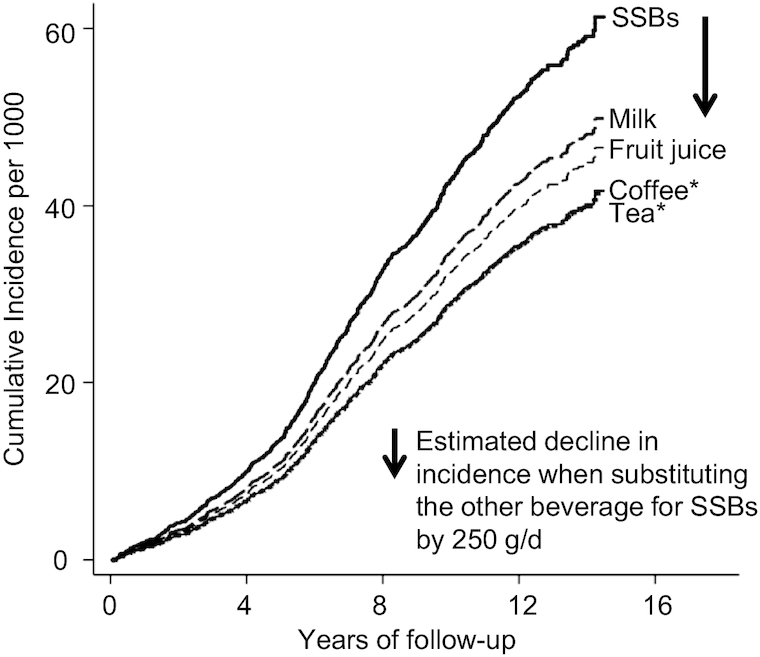
Prospective associations of substituting alternative beverages for SSBs with incidence of type 2 diabetes in the EPIC–InterAct case–cohort study. Cumulative incidence curves are displayed for adults consuming >250 g/d of SSB and adults modeled to replace SSB with 1 alternative beverage (milk, fruit juice, coffee, or tea) by 250 g/d: those for coffee and tea were almost identical. Baseline hazards of the subcohort and regression estimates (Table 3) derived from multivariable-adjusted Prentice-weighted Cox regression including the 5 variables simultaneously, demographic factors (recruitment centers, age, and sex), education, marital status, hormone replacement therapy, menopausal status, history of oral contraceptive use, hypertension, dyslipidemia, family history of diabetes, prevalent diseases (coronary heart disease or stroke), BMI, waist circumference, smoking, physical activity, alcohol consumption, dietary supplement use, and dietary consumption (total energy intake, vegetables, fruits, nuts, cheese, yogurt, red meats, processed meats, fish, confectionary, and cereals). *P < 0.001. EPIC, European Prospective Investigation into Cancer and Nutrition; SSB, sugar-sweetened beverage.

Modeling comparative associations of beverage consumption with T2D, substituting coffee or tea for SSBs was estimated to lower T2D incidence significantly (**Figure 1**, **Table 3**). In analyses adjusted for potential confounders and adiposity measures, substituting coffee for SSBs by 250 g/d was estimated to lower the incidence of T2D by 21% (95% CI: 12%, 29%) or lower cases by 12.0 (95% CI: 5.0, 20.0) per 10,000 person-years of adults consuming SSBs ≥250 g/d. Similarly, substituting tea for SSBs was estimated to lower T2D incidence by 22% (95% CI: 15%, 28%) or T2D events by 11.0 (95% CI: 2.6, 20.0) per 10,000 person-years. When considering 5- and 20-y follow-up instead of 10 y, RDs were halved and doubled, respectively, as would be expected (**Supplemental Table 6**). The other estimates of substitution effects were not significant: as exception, the substitution of coffee or tea for milk was significantly associated with lower incidence (Table 3).

**TABLE 3:** Prospective associations of beverage consumption with incident type 2 diabetes, modeled to estimate incidence rates if participants substituted 1 beverage for the other: EPIC-InterAct case–cohort study (*n* = 27,662)^1^

	Incidence per 10,000 person-years^2^	Alternative beverages substituted for the beverage in the row (by 250 g/d)				
Beverage estimated to be substituted for an alternative beverage (per 250 g/d)		Sugar-sweetened beverages	Fruit juice	Milk	Coffee	Tea
HR (95% CI)^3^						
Sugar-sweetened beverages	39.5	1.00 (reference)	0.89 (0.74, 1.07)	0.91 (0.82, 1.02)	0.79 (0.72, 0.88)	0.78 (0.72, 0.85)
Fruit juice			1.00 (reference)	1.03 (0.9, 1.18)	0.89 (0.83, 0.95)	0.89 (0.76, 1.05)
Milk				1.00 (reference)	0.85 (0.78, 0.92)	0.82 (0.74, 0.91)
Coffee					1.00 (reference)	1.02 (0.94, 1.11)
Tea						1.00 (reference)
Rate differences (95% CI) per 10,000 person-years in adults with ≥250 g/d for each beverage^4^						
Sugar-sweetened beverages	48.3	0.0 (reference)	−5.8 (−16.0, 4.4)	−2.0 (−8.5, 4.4)	−12.0 (−20.0, −5.0)	−11.0 (−20.0, −2.6)
Fruit juice	25.2	4.1 (−3.9, 12.0)	0.0 (reference)	2.2 (−2.8, 7.2)	−3.4 (−8.2, 1.4)	−2.8 (−7.9, 2.4)
Milk	41.3	3.6 (−5.2, 12.0)	−2.8 (−10.0, 4.4)	0.0 (reference)	−8.2 (−13, −3.7)	−7.3 (−13.0, −1.4)
Coffee	38.4	12.0 (2.5, 21.0)	5.4 (−2.2, 13.0)	8.2 (3.7, 13.0)	0.0 (reference)	0.9 (−4.6, 6.5)
Tea	28.5	11.0 (0.6, 21.0)	4.5 (−4.0, 13.0)	7.3 (1.4, 13.0)	−0.9 (−6.5, 4.6)	0.0 (reference)

1 12,333 cases were evaluated, along with the subcohort of the total (774 cases/192,287 person-years). Prentice-weighted Cox proportional hazard regression was modeled for each country. Country-specific estimates were pooled by multivariable random-effects meta-analysis. EPIC, European Prospective Investigation into Cancer and Nutrition.

2 Calculated from the subcohort.

3 In each model, all beverages were mutually adjusted for each other. Demographic covariates included recruitment centers, age, and sex. Further adjustment for potential confounders included education, marital status, hormone replacement therapy, menopausal status, history of oral contraceptive use, hypertension, dyslipidemia, family history of diabetes, prevalent diseases (coronary heart disease and stroke), BMI, waist circumference, smoking, physical activity, alcohol consumption, dietary supplement use, and dietary consumption (total energy intake, vegetables, fruits, nuts, cheese, yogurt, red meats, processed meats, fish, confectionary, and cereals).

4 Analysis was performed by estimating incidence rate of adults consuming ≥250 g/d of the beverage of each row and by estimating rate differences representing effects of replacing the beverage of each row with the beverage of each column. The second column presents crude rates per 10,000 person-years of adults consuming ≥250 g/d of each beverage.

In analyses examining subtypes of beverages among subsets, consumption of sweetened milk beverages (separated from SSBs) was positively associated with incident T2D with an HR of 2.56 (95% CI: 1.04, 6.29) per 250 g/d (**Supplemental Table 7**). Other subgroups of beverages (decaffeinated coffee, vegetable juice, ASBs, and water) were not significantly associated with incident T2D. The HR for vegetable juice was not estimated precisely (95% CI: >10). Substituting decaffeinated coffee, ASBs, or water for SSBs was estimated to lower T2D incidence by 23% (95% CI: 15%, 30%), 22% (95% CI: 17%, 26%), or 13% (95% CI: −1.0%, 24%), respectively. Heterogeneity by country was moderate to substantial overall. For example, *I*^2^ varied from 39.7% for tea to 77.3% for replacing SSBs with another type of beverage (**Supplemental Figure 3**). The heterogeneity was not explained by average age, proportion of men or women, average BMI, or absolute incidence (*P* > 0.05 in meta-regression). Of prespecified variables, sex was identified to be a significant effect modifier for fruit juice (*P*-interaction =  0.02; *P* > 0.2 for others; Supplemental Figure 3). In men, but not in women, substituting fruit juice for SSBs was estimated to reduce T2D risk (HR: 0.69; 95% CI: 0.53, 0.90). This association in men and the heterogeneity by sex were not significant (*P* > 0.2 for each) in a post hoc analysis censoring events during the first 6 y of follow-up.

## Discussion

Substituting consumption of coffee or tea for consumption of SSBs was associated with an ∼20% lower incidence of T2D across 8 European populations. In secondary analyses, substitution of decaffeinated coffee or ASBs for SSBs was also associated with lower incidence of T2D. By contrast, fruit juice and milk appeared unlikely to be suitable alternatives to SSBs for the prevention of T2D. Our study suggests the potential benefits of alternative beverages to SSBs for the primary prevention of T2D.

Our analysis provides the first evidence for populations of multiple European countries that coffee consumption was inversely associated with T2D incidence, whereas the association has been well established in individual countries across global regions (7); consumption of water or vegetable juice was not significantly associated with T2D incidence; and consumption of sweetened milk beverages was significantly positively associated with T2D incidence. Potential effects of substituting 1 beverage for another on T2D have been examined in only a few studies in the United States or the United Kingdom (16–18). We were unable to distinguish between sweetened and unsweetened tea or coffee, whereas EPIC-Norfolk evaluated detailed 7-d dietary records and highlighted positive associations of sweetened coffee or tea with T2D risk (18). Available evidence from this study and others indicates that T2D risk may be reduced by substituting unsweetened coffee or tea for SSBs but not by using fruit juice or milk as the replacement beverages.

Our secondary analysis of substituting ASBs for SSBs by 1 serving/d in the subset produced a stronger estimate (Supplemental Table 7) than that from the Nurses’ Health Study (HR: 0.95; 95% CI: 0.90, 0.98) (16) or the EPIC-Norfolk study (its study population partly overlapped with the population in EPIC-InterAct) (HR: 0.95; 95% CI: 0.80, 1.08) (17, 18). The heterogeneity between these results may have reflected different degrees of population-specific residual confounding (38, 39) or the use of different dietary assessment methods. Accounting for the heterogeneity and the uncertainty of potential adverse effects of ASBs on appetite, gut microbiota, and metabolic risks (38), evidence remains weak for the benefit of substituting ASBs for SSBs for T2D prevention. In modeling substitution of water for SSBs, the potential reduction in T2D incidence appeared quantitatively similar to those in EPIC-Norfolk (HR: 0.86; 95% CI: 0.74, 0.99) (no overlapping population with EPIC-InterAct) (18) and the Nurses’ Health Study (HR: 0.93; 95% CI: 0.89, 0.97) (16). Despite the consistency, results on consumption of water, as well as ASBs, should be interpreted cautiously partly because of residual confounding due to health consciousness. Based on our findings and other evidence, further research using controlled clinical studies and population-based studies is needed to better understand the efficacy of consumption of ASBs and water (or hydration) on the development of T2D (4, 40). Although this is beyond the scope of our study, ASBs and water may serve as favored alternatives (4, 40, 41) to SSBs because ASBs and water have low or zero energy content, and SSBs, ASBs, and water share a similar context of consumption as cold beverages. Effectiveness is likely to be related to such practical considerations, and relevant behaviors remain to be studied as well.

Our study indicated that milk consumption was positively associated with T2D but not after accounting for under- or overreporters of dietary consumption, measurement error, or reverse causation. These findings and prior nonsignificant findings in meta-analyses of low-fat or high-fat milk and T2D (23) indicate that milk is unlikely to be a healthy alternative to SSBs for the prevention of T2D. Clinical trials have suggested a benefit of milk consumption through its insulinogenic and antioxidative properties (42) with diversity in comparators (e.g., water or other beverages) and types of milk (e.g., full-fat or skim). Sweetened milk beverages have been little studied but are likely to be harmful based on biological plausibility related to added sugars (18) and this study using FFQs and the EPIC-Norfolk study using 7-d food records (18). In summary, specific types of milk remain to be evaluated, but consumption of milk or milk beverages is unlikely to contribute to the primary prevention of T2D.

Policy implications from this work deserve discussion. Our findings indicate the possible benefit of explicitly recommending alternatives to SSBs. This corroborates with the principle of beverage guidelines proposed in 2006 for the US population (4). The importance of such guidelines has been further supported by our estimates of T2D risk differences in the order of 10s per 10,000 person-years. This indicates that a benefit for individuals may be too small, but a population-level benefit could be meaningful, particularly in countries in which prevalence of T2D is alarming (43) and millions of adults are consuming SSBs [e.g., 50% of adults in the United States (5) and up to two-thirds in European countries (10)]. Our findings for consumption of milk and sweetened milk beverages also provide an implication for dietary guidelines. Dairy consumption has been widely recommended in federal dietary guidelines as a source of calcium, vitamin D, and other nutrients, and this recommendation has been questioned (44). By contrast, our finding does not support a presumed benefit, whereas sweetened milk is likely to be harmful at least in the context of the primary prevention of T2D. Further clinical, modeling, and policy research are warranted to explore the utility of explicitly recommending substitution of specific beverages for SSBs in diverse populations.

Despite no direct evidence, our work should stimulate further research on the effectiveness and efficacy of substituting alternative beverages for SSBs. For individuals who are at high risk of T2D and consume SSBs habitually, advice to consume other beverages instead of SSBs is reasonable to consider, rather than advice to reduce SSBs solely. Its benefit has been indicated in a trial demonstrating improvement of a diet by substituting ASBs or water for SSBs (45). Reducing SSB consumption may be easier to achieve than modifying food consumption because SSBs are often consumed in isolation rather than in combination after being cooked, for instance, with other foods. To strengthen these considerations with empirical evidence, behavioral effects of promoting consumption of healthy alternatives to SSBs should be established in future intervention studies.

EPIC-InterAct had the strengths of longitudinal design; a large number of T2D cases adjudicated in a standardized manner (19); the availability of covariates; 24-h recall dietary data in the subset; and a wide variety of beverage types, including water and milk beverages for which little evidence was available to date. A number of limitations of this study typical of observational research exist and limit a causal inference for a dietary effect on disease incidence. Thus, the substitution effects should be considered as estimates modeled from observational evidence. Relevant limitations include residual confounding due to unmeasured or imprecisely measured confounders. Although our calibration for measurement errors indicated robustness of our main conclusion, measurement errors were likely to exist heterogeneously across countries in estimating absolute amounts of beverage consumption and in estimating habitual consumption because dietary exposures were measured only at baseline. Bias during the follow-up could occur; for example, an increase in SSB consumption over years (10, 11) could have widened between-individual variance of SSB consumption and made HRs per 1 serving/d overestimated. Misclassification is also possible because InterAct (and many previous studies) had no data on sports drinks, sweetened coffee or tea, and other subtypes, although these subtypes of beverages may increase the risk of T2D (3, 4, 18, 46). Availability of data was limited on preparation methods for beverages; for instance, populations in Italy and the United Kingdom prepare (brew) coffee differently, which may have differential biological effects and contribute to heterogeneity in prospective associations. Last, generalizability is limited because we primarily studied white adults in Europe (7, 8). Because tea, coffee, SSBs, ASBs, and water are consumed globally (2, 3) in diverse cultural and culinary settings, future work is warranted to characterize healthy alternatives to SSBs in different regions of the world.

In conclusion, across 8 European countries, SSB consumption was positively associated, and consumption of coffee or tea was inversely associated, with the risk of developing T2D. In our modeling of substitution effects of different beverages, ∼20% of risk was estimated to be reduced by substituting coffee or tea for SSBs. This work provides implications for the primary prevention of T2D by reducing SSB consumption and increasing consumption of healthier beverage options.

## Supplementary Material

nxz156_Supplemental_File

## Notes

The InterAct project was funded by the EU FP6 program (LSHM_CT_2006_037197). InterAct investigators acknowledge funding from the following agencies: Medical Research Council Epidemiology Unit Core Support (MC_UU_12015/1, MC_UU_12015/5); National Institute for Health Research, Biomedical Research Centre Cambridge: Nutrition, Diet, and Lifestyle Research Theme (IS-BRC-1215-20014); the German Federal Ministry of Education and Research, the German Center for Diabetes Research, the State of Brandenburg, Germany; Netherlands Agency grant IGE05012 and an Incentive Grant from the Board of the University Medical Center Utrecht (Netherlands); Dutch Ministry of Public Health, Welfare, and Sports, Netherlands Cancer Registry, LK Research Funds, Dutch Prevention Funds, Dutch ZON (Zorg Onderzoek Nederland), World Cancer Research Fund, and Statistics Netherlands; Swedish Research Council, Novo Nordisk, Swedish Heart Lung Foundation, and Swedish Diabetes Association; Danish Cancer Society; Deutsche Krebshilfe; Associazione Italiana per la Ricerca sul Cancro; Asturias Regional Government; Navarra Regional Government, Health Research Fund of the Spanish Ministry of Health; CIBER Epidemiología y Salud Pública, Spain; Murcia Regional Government (grant 6236); and AIRE-ONLUS Ragusa, AVIS-Ragusa, and Sicilian Regional Government.

Author disclosures: FI, MBS, SJS, MG, DR, BB, ES-F, EA, LA, DA, HB, CD, GF, PWF, HF, PJ, RK, K-TK, TK, FRM, GM, M-DC, PMN, KO, VMP, SP, AP-C, JRQ, FR, MR-B, OR, IS, MS, AMWS, AT, TYNT, RT, LETV, HAW, CL, ER, NGF, and NJW, no conflicts of interest.

Supplemental Figures 1–3, Supplemental Tables 1–7, and Supplemental Methods are available from the “Supplementary data” link in the online posting of the article and from the same link in the online table of contents at https://academic.oup.com/jn/.

NGF and NJW contributed equally to this work.

Abbreviations used: ASB, artificially-sweetened beverage; EPIC, European Prospective Investigation into Cancer and Nutrition; HbA1c, glycated hemoglobin; hsCRP, high-sensitivity C-reactive protein; RD, rate difference; SSB, sugar-sweetened beverage; T2D, type 2 diabetes.
