# Supplementary material for: Estimated Substitution of Tea or Coffee for Sugar-Sweetened Beverages Was Associated with Lower Type 2 Diabetes Incidence in Case–Cohort Analysis across 8 European Countries in the EPIC-InterAct Study
Source: J Nutr. 2019 Aug 8;149(11):1985–93. doi: 10.1093/jn/nxz156 (PMC6825826; doi:10.1093/jn/nxz156)
Supplement: nxz156_Supplemental_File [file nxz156_supplemental_file.pdf]

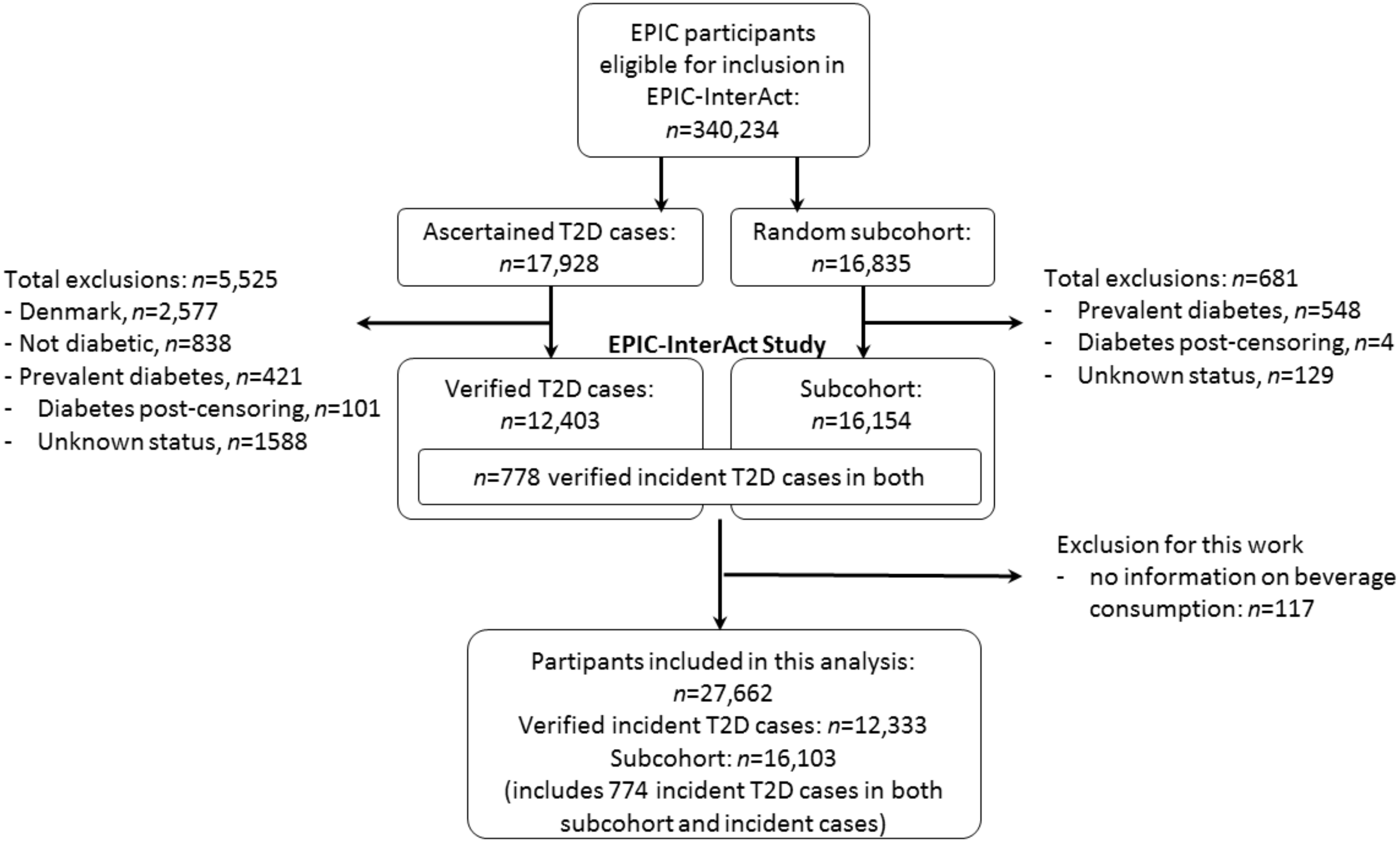

Supplemental Figure 1. The case-cohort design of the European Prospective Investigation into Cancer and Nutrition InterAct (EPIC-InterAct). The original EPIC study recruited 519,978 participants from 10 countries. In 8 countries participating EPIC-InterAct, 455,680 participants were recruited and 346,055 provided biological samples. After we further excluded 109,625 without available stored blood and 5,821 without information on diabetes status, the study base of 340,234 participants was formulated in InterAct.

Supplemental Table 1. Type 2 diabetes ascertainment and verification sources in the European Prospective Investigation into Cancer and Nutrition InterAct[1]

	France	DK[2]	Italy					Spain					UK[2]		NL[2]		Germany		Sweden	
**Sources**	All[2]	All[2]	Florence	Varese	Ragusa	Turin	Naples	Asturias	Granada	Murcia	Navarra	San Sebastian	Cambridge	Oxford	Bilthoven	Utrecht	Heidelberg	Potsdam	Malmo	Umea
**Ascertainment**																				
Self-report of diabetes and/ or date of diagnosis	x		x	x			x	x	x	x	x	x	x	x	x	x	x	x		
Self-report of diabetes medication	x		x				x	x					x	x				x		
Self-report of diabetes-related diet	x																	x		
External evidence of diabetes medication (primary care/pharmacy registers, reported by pharmacist)	x	x	x		x	x		x	x	x	x	x								x
Diabetes code on death certificate			x			x		x	x	x	x	x	x	x			x	x		
External evidence of diabetes related hospital visits (inpatient and/or outpatient)		x	x	x	x	x	x	x	x	x	x	x	x		x	x	x			
Self-report of diabetes related hospital visit (inpatient and/or outpatient)	x							x												
External evidence of diabetes from registers (general practitioner [GP] or non-profit)		x	x		x	x		x		x	x	x	x				x	x	x	x
External evidence of diabetes from GP report or medical records																	x	x		
Laboratory results/register data specific to diabetes		x										x							x	x
Self-report of laboratory results indicating diabetes																x				
**Verification**																				
Self-report of diabetes and/or date of diagnosis	x		x	x	x		x	x	x	x	x	x	x	x	x	x	x			
Self-report of diabetes medication	x		x	x	x			x					x	x						
Self-report of diabetes-related diet	x																			
External evidence of diabetes medication (primary care/ pharmacy registers, reported by pharmacist)	x	x	x	x	x	x		x	x	x	x	x				x				x
Diabetes code on death certificate			x	x	x	x	x	x	x	x	x	x	x	x			x			
External evidence of diabetes-related hospital visits (inpatient and/or outpatient)		x	x	x	x	x	x	x	x	x	x	x	x		x	x	x			
Self-report of diabetes-related hospital visit (inpatient and/or outpatient)								x												
External evidence of diabetes from registers (general practitioner [GP] or non-profit)		x				x		x	x	x	x	x	x				x	x	x	x
External evidence of diabetes from GP report or medical records								x	x	x	x	x	x	x	x	x	x	x		
Laboratory results/register data specific to diabetes		x	x				x	x			x	x	x						x	x
Self-report of laboratory results indicating diabetes	x		x													x				

[1] Adopted from Langenberg et al.(1)
[2] DK, Denmark; NL, the Netherlands; UK, the United Kingdom. France recruited participants at five sites classified as Ile-de-France, north west, north east, Rhone-Alpes/Auvergne, Provence/Languedoc, and South-West. Denmark recruited participants at two sites: Aarhus and Copenhagen.

Supplemental Table 2. Baseline consumption and classification of different beverages: the subcohort in the European Prospective Investigation into Cancer and Nutrition InterAct case-cohort study (*n*=16,103) [1]

**Beverage categories**	*n*	% of adults ≥250 g/day	Mean±SD g/day	Beverage items included[1]	Comments on availability and classification
**Evaluated in the primary analysis**[2]					
Sugar-sweetened beverages	16,103	6.2	54.5±105	• Carbonated/soft/isotonic drinks, diluted syrups, sweetened or unspecified, containing >2 g carbohydrates/100 g<br>• Milk beverages	Centers (except Naples) in Italy and Umea in Sweden did not separate out artificially sweetened beverages Milk beverages were assessed in limited regions (see the row of 'milk beverages')
Fruit juice	16,103	4.9	59.2±101	• Juice not specified, non-citrus juice, mixed juice, without added sugars, nectors and juices from concentrate<br>• Citrus juices<br>• Vegetable juices	Spain, the United Kingdom, Naples in Italy, and Umea in Sweden did not disaggregate the two items.
Milk	16,103	32.7	209±203	• Milk	French, Italy (except Asturias), the United Kingdom, and Sweden did not separate out sweetened milk beverages
Coffee	16,103	52.5	381±372	• Coffee (not specified)<br>• Caffeinated coffee<br>• Decaffeinated coffee	France, Ragusa and Naples in Italy, Umea in Sweden, and Denmark did not separate caffeinated and decaffeinated coffee
Tea	16,103	20.8	152±282	• Tea	
**Included in the primary analysis as a part of main beverages, but separately evaluated in secondary analysis**[2]					
Artificially sweetened beverages	13,324	2.2	23.7±97.9	• Carbonated/soft/isotonic drinks, diluted syrups, artificially sweetened or unsweetened, containing <2 g carbohydrates/100 g	French (except for Naples) and Umea in Sweden did not assess this variable.
Sweetened milk beverages	8533	0.5	12.5±41.4	• Sweetened milk beverages	Not assessed in French, Italy (except Asturias), the United Kingdom, and Sweden
De-caffeinated coffee	11,824	4.3	33.8±122	• Coffee, decaffeinated	Not assessed in France, Ragusa and Naples in Italy, Umea in Sweden, and Denmark
Vegetable juice	9,140	0.1	2.7±16.7	• Vegetable juices	Not assessed in Spain, the United Kingdom, Naples in France, and Umea in Sweden
**Not included in the primary analysis, but evaluated in secondary analysis**					
Water	9,209	61.6	496±488	• Water	Not assessed in Italy, Spain, and the United Kingdom. Not assessed for subtypes (tap water, distilled water, carbonated water, or flavoured water)

[1] *n*=16,103 from the sub-population originally randomly sampled from the European Prospective Investigation into Cancer and Nutrition InterAct cohort (Supplemental Figure 1). Details for dietary measurements were reported previously(2,3)

[2] Results from the primary analysis are presented in Table2 and Table 3; and from the secondary analysis, Supplementary Table 7.

Supplemental Table 3. Baseline characteristics by beverage consumption: the subcohort of the European Prospective Investigation into Cancer and Nutrition InterAct case-cohort study (n=16,103)[1]

	Sugar-sweetened beverages		Fruit juice		Milk		Coffee		Tea	
	<250 g/day	≥250 g/day	<250 g/day	≥250 g/day	<250 g/day	≥250 g/day	<250 g/day	≥250 g/day	<250 g/day	≥250 g/day
% of 16,103 participants	94%	6.2%	95%	4.9%	67%	33%	48%	52%	79%	21%
% of non-consumers[2]	46%		25%		7.9%		6.9%		47%	
Age, years	53±9.0	50±11*	52±9.1	51±10*	52±8.8	53±10*	51±9.2	53±9.1*	52±8.9	54±9.7*
Sex, women	63	48*	62	65	62	62	63	61	61	67*
Education, ≥high school	21	17	20	30*	22	19*	17	23*	19	28*
Currently employed	51	56*	50	66*	54	46*	36	65*	47	66*
Current smokers	26	30	26	23	26	25	22	29*	28	18*
Physical activity, ≥moderately active	42	49*	42	50*	43	43	36	49*	41	50*
Family history of diabetes‡	20	20	17	20	22	21	18	18	20	21
Prevalent conditions										
Hypertension	19	18	18	25*	20	16*	19	18	19	17*
Dyslipidemia	18	19	18	25*	20	15*	21	16*	19	16*
Cardiovascular disease	2.4	2.2	2.3	3.2	2.3	2.5	2	2.6	2.2	2.9
Dietary supplement use	3.1	3	3	3.7	2.9	3.3	2.5	3.5*	2.9	3.6
Dietary consumption										
Energy, mega Joules/day	8.9±2.8	10.5±3.3*	8.9±2.8	9.8±3.1*	8.7±2.8	9.5±2.9*	9.1±2.9	8.9±2.7*	9±2.9	8.8±2.7*
Alcohol, servings/day	0.95±1.3	0.89±1.4	0.96±1.3	0.82±1.1	1.06±1.4	0.72±1.1*	0.94±1.4	0.96±1.2	0.99±1.4	0.8±1.1*
Fibre, g/day	23±8	23±9	23±8	24±8	23±8	23±8*	23±8	22±8*	23±8	24±8*
Vegetables, g/day	186±120	160±115*	186±121	153±98*	181±118	191±124*	210±132	161±103*	181±121	196±117*
Fruits, g/day	237±191	208±191*	237±192	197±164*	234±195	237±183	285±213	189±155*	239±196	220±168*
Processed meat, g/day	36±32	48±40*	36±32	52±43*	38±34	35±31*	33±31	40±34*	38±34	30±28*
Yoghurt, g/day	62±88	69±95	62±88	83±96*	62±87	65±91	47±73	77±98*	60±87	74±94*
Confectionary, g/day	24±49	36±75*	25±52	24±34	24±49	27±54*	18±39	31±59*	23±50	32±55*
Ratio of energy intake to energy requirement	0.91±0.29	1.00±0.35*	0.91±0.29	0.98±0.36*	0.89±0.29	0.97±0.30*	0.95±0.31	0.88±0.28*	0.92±0.30	0.89±0.28*
BMI, kg/m^2	10.9	5.0*	10.7	6.7*	12.4	6.6*	9.3	11.6*	10.6	10.2
Waist circumference, cm	26±4	26±4	26±4	25±4*	26±4	26±4	27±4	26±4*	26±4	25±4*
Hemoglobin A1c, mmol/mol	86±13	88±13*	86±13	85±13*	86±13	86±13	87±13	85±13*	87±13	83±12*
Triglycerides, mmol/L	36±5	36±6	36±5	36±5	36±5	37±5*	36±5	36±5*	36±5	36±4*
HDL-cholesterol, mmol/L	1.3±0.9	1.5±1*	1.3±0.9	1.5±1*	1.3±0.9	1.4±0.9*	1.3±0.9	1.4±0.9*	1.3±0.9	1.4±0.8*
C-reactive protein, mg/L	1.5±0.4	1.4±0.4*	1.5±0.4	1.5±0.4	1.5±0.4	1.5±0.4*	1.5±0.4	1.5±0.4	1.5±0.4	1.5±0.4*

[1] For continuous and categorical variables, respectively, means (standard deviations) and proportions are presented. Case-cohort design was implemented in European Prospective Investigation into Cancer and Nutrition-InterAct (EPIC-InterAct). Statistics from the subcohort are used in analyses for this table. Asterisk next to a value (*) is presented for the variable with statistically significant association with the beverage (P>0.05).

[2] Proportions of adults who reported no or a minimal level of consumption in a dietary questionnaire/history among 16,103 participants.

[3] not available in two research centers and thus evaluated among 8,850 adults in the subcohort.

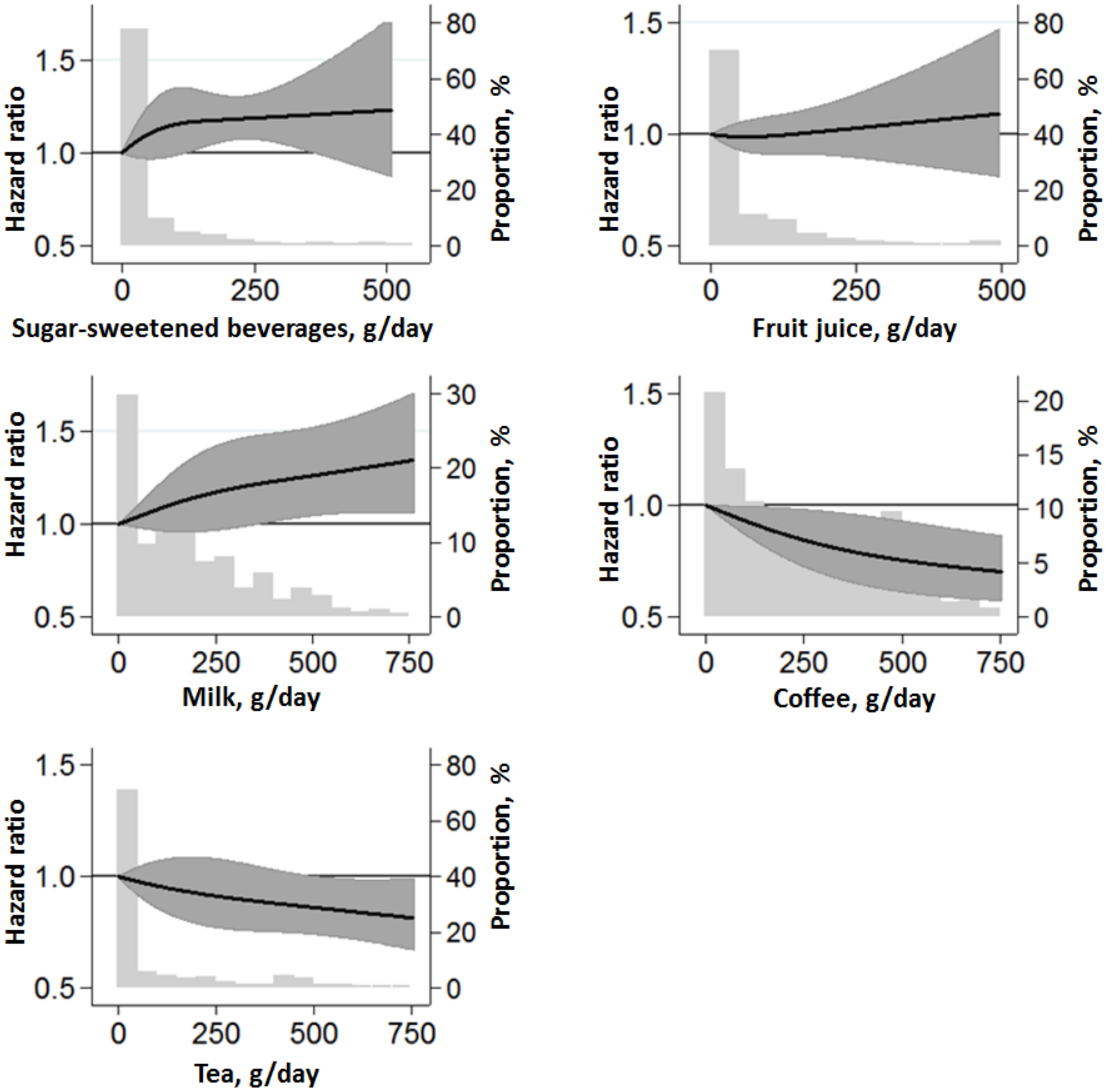

Supplemental Figure 2. Prospective associations between consumption of each type of beverages and incident type 2 diabetes (*n*=27,662). Each solid line and grey curved area represents hazard ratio and 95% confidence interval (left axis). Bars represent histogram (proportion representing % of adults, right axis) estimated in the subcohort. Country-specific estimates from Prentice-weighted Cox regression with a restricted cubic spline function were obtained first and then pooled by multivariable random-effects meta-analysis. In each model, all beverages were mutually adjusted (a linear term, when used as a covariate). Each model was adjusted for age (underlying time variable), recruitment centers, sex, education, marital status, hormone replacement therapy, menopausal status, history of oral contraceptive use, hypertension, dyslipidemia, family history of diabetes, prevalent cardiovascular diseases, smoking, physical activity, alcohol consumption, dietary supplement use, dietary consumption (total energy intake, vegetables, fruits, nuts, cheese, yoghurt, red meats, processed meats, fish, confectionary, and cereals), BMI, and waist circumference. Non-linear associations were not significant for any of the variables (*P* for non-linearity>0.2). Linear relationships were significant (*P*<0.05), except for fruit juices, as reported in Table 2.

Supplemental Table 4. Prospective associations of beverage consumption with incidence of type 2 diabetes: sensitivity analysis and analysis for potential mediators in the European Prospective Investigation into Cancer and Nutrition – InterAct case-cohort study (*n*=27,662)[1]

Scenario	Hazard ratio (HR) (95% confidence intervals) per 250 g/day [2]				
	SSB	Fruit juice	Milk	Coffee	Tea
Adjusted for potential confounders and adiposity measures	1.18 (1.08, 1.28)	1.06 (0.96, 1.17)	1.10 (1.02, 1.19)	0.91 (0.89, 0.94)	0.93 (0.87, 0.98)
Substitution for SSB per 250 g/day	1.0 (reference)	0.89 (0.74, 1.07)	0.91 (0.82, 1.02)	0.79 (0.72, 0.88)	0.78 (0.72, 0.85)
Not winsorized	1.13 (1.07, 1.20)	1.05 (0.97, 1.15)	1.12 (1.04, 1.20)	0.92 (0.90, 0.95)	0.95 (0.90, 1.01)
Substituted for SSB per 250 g/day	1.0 (reference)	0.90 (0.74, 1.08)	0.91 (0.81, 1.02)	0.79 (0.72, 0.88)	0.78 (0.72, 0.85)
Exclusion of under- and over-reporters	1.13 (0.99, 1.30)	1.09 (0.97, 1.23)	1.08 (0.99, 1.17)	0.89 (0.85, 0.93)	0.91 (0.84, 0.98)
Substituted for SSB per 250 g/day	1.0 (reference)	0.96 (0.75, 1.23)	0.94 (0.79, 1.11)	0.79 (0.67, 0.93)	0.79 (0.67, 0.93)
Censoring events diagnosed during the first six years	1.15 (1.03, 1.28)	1.09 (1.00, 1.18)	1.06 (0.96, 1.18)	0.92 (0.90, 0.95)	0.92 (0.85, 0.99)
Substituted for SSB per 250 g/day	1.0 (reference)	0.91 (0.75, 1.10)	0.90 (0.78, 1.04)	0.82 (0.73, 0.93)	0.79 (0.69, 0.91)
Multiple imputation (20 datasets)	1.20 (1.08, 1.34)	1.03 (0.91, 1.17)	1.11 (1.01, 1.23)	0.91 (0.87, 0.95)	0.92 (0.86, 0.98)
Substituted for SSB per 250 g/day	1.0 (reference)	0.86 (0.70, 1.05)	0.93 (0.82, 1.04)	0.75 (0.66, 0.86)	0.77 (0.70, 0.84)
Log transformed (base=2)	1.21 (1.10, 1.33)	1.05 (0.94, 1.17)	1.14 (1.03, 1.27)	0.84 (0.78, 0.91)	0.89 (0.81, 0.97)
Halving SSB and doubling the other beverages	1.0 (reference)	0.87 (0.72, 1.04)	0.91 (0.79, 1.05)	0.71 (0.62, 0.80)	0.73 (0.65, 0.81)
Corrected for regression dilution[3]	1.44 (1.04, 2.01)	2.32 (1.21, 4.44)	1.10 (0.94, 1.28)	0.89 (0.84, 0.95)	0.90 (0.87, 0.93)
Substituted for SSB per 250 g/day	1.0 (reference)	0.73 (0.49, 1.10)	0.78 (0.52, 1.15)	0.62 (0.47, 0.84)	0.69 (0.51, 0.94)
Assigned 250 g to SSB, 150 g to the others per serving	1.18 (1.08, 1.28)	1.02 (0.95, 1.10)	1.06 (1.00, 1.13)	0.95 (0.92, 0.97)	0.95 (0.92, 0.99)
Substituted for SSB per one serving/day	1.0 (reference)	0.86 (0.74, 0.99)	0.89 (0.81, 0.98)	0.79 (0.71, 0.88)	0.80 (0.74, 0.87)
Additionally adjusted for potential mediators[4]	1.14 (1.03, 1.26)	1.01 (0.87, 1.17)	1.03 (0.93, 1.15)	0.93 (0.90, 0.97)	0.93 (0.86, 1.01)
Substituted for SSB per 250 g/day	1.0 (reference)	0.91 (0.72, 1.16)	0.89 (0.79, 1.00)	0.84 (0.75, 0.94)	0.82 (0.73, 0.91)

[1] The different analytic options and potential mediation by physiological factors§ were tested separately. For substitution between beverages, that for SSB is presented; other results were not materially different from the main results (Table 2).

[2] Top two rows were considered as primary results. Prentice-weighted Cox models were fit separately to data from each country. Country-specific estimates were pooled by random-effects meta-analysis. All beverages in each model were mutually adjusted. Demographic covariates included recruitment centers, age, and sex. Further adjustment for potential confounders included education, marital status, hormone replacement therapy, menopausal status, history of oral contraceptive use, hypertension, dyslipidemia, family history of diabetes, prevalent diseases (coronary heart disease and stroke), smoking, physical activity, alcohol consumption, dietary supplement use, and dietary consumption (total energy intake, vegetables, fruits, nuts, cheese, yoghurt, red meats, processed meats, fish, confectionary, and cereals)..

[3] Potential bias due to regression dilution was corrected by applying a multivariable formula of $\beta_{calibrated}=\beta_{uncalibrated}\times(\sum_{total}-\sum_{error})^{-1}\sum_{total}$, where $\beta_{calibrated}$ and $\beta_{uncalibrated}$ is a vector for calibrated and uncalibrated regression coefficients for the main beverages, respectively; and $\sum_{total}$ and $\sum_{error}$ represent total and error variance-covariance matrix, respectively, of beverage consumption estimated by FFQ (please see Supplemental Table 5 for $\sum_{error}$). Regression calibration was conducted in each country, and calibrated regression coefficients and related variance estimates were meta-analyzed and converted to hazard ratios and those for potential substitution effects.

[4] Physiological markers were adjusted for as potential mediators: triglycerides, high-density lipoprotein cholesterol, hemoglobin A1c, and C-reactive protein.

Supplemental Table 5. Beverage consumption estimated from food frequency questionnaires (FFQs) and from 24-hour recall and potential regression dilution in prospective associations of beverage consumption with incident type 2 diabetes: calibration analysis in the European Prospective Investigation into Cancer and Nutrition – InterAct case-cohort analysis[1]

Beverage	Mean±SD		Variance-covariance matrix used for multivariable-regression calibration				
	FFQ	24 h recall	Sugar-sweetened beverages	Fruit juice	Milk	Coffee	Tea
Sugar-sweetened beverages	0.24±0.45	0.21±0.68	0.41*	0.04	0.01	0.01	0.01
Fruit juice	0.24±0.41	0.26±0.67	0.04	0.46*	-0.02	-0.04	-0.03
Milk	0.87±0.84	0.81±0.97	0.01	0.00	0.60*	-0.03	0.01
Coffee	1.54±1.51	1.70±1.75	-0.01	-0.01	-0.01	0.63*	-0.04
Tea	0.60±1.13	0.59±1.23	-0.01	-0.01	-0.01	-0.06	0.50*

[1] To examine errors in FFQ, adults with potential systematic errors observed as under- or over-reporters were excluded, resulting in 22,860 adults (from 27,662 adults) with at least FFQ and 1950 adults (from 2,347 adults) with both FFQ and 24-hour recall data. Crude averages were calculated across the eight countries for each of descriptive statistics and variance matrix. The variance matrix is an inverse matrix of $(\sum_{total}-\sum_{error})^{-1}\sum_{total}$ for use of multivariable regression calibration: $\beta_{calibrated}=\beta_{uncalibrated}\times(\sum_{total}-\sum_{error})^{-1}\sum_{total}$ (See Supplemental Table 4). Thus, the diagonal cells (*) ranging from 0.41 to 0.50 in this table indicates the magnitude of regression dilution. The calculation was performed for each country. After country-specific multivariate calibration for estimates from Cox proportional hazard analysis, calibrated estimates were meta-analyzed. The variance matrices were obtained after standardizing variance of estimates from FFQ to variance of estimates from 24-hour recalls, considering difference in variances or standard deviations between FFQ and 24-hour recall as a source of bias; and after adjustment for potential confounders including age sex, smoking status, education, occupation status, marital status, physical activity levels, dietary consumption estimated from FFQ (energy intake, fruits, vegetables and alcohol), prevalent cardiovascular diseases, body mass index, and additional variables related to 24-hour recall data (energy intake, weekend/weekdays, or seasons).

Supplemental Table 6. Incidence rate difference in type 2 diabetes, associated with substitution between two types of beverages for different years of follow-up or for 10 years among consumers of a specific beverage: the European Prospective Investigation into Cancer and Nutrition – InterAct case-cohort analysis (*n*=27,662).

Beverages	Absolute Incidence[1]	Alternative beverages by 250 g/day[2]				
		Sugar-sweetened beverages	Fruit juice	Milk	Coffee	Tea
Follow-up for 5 years						
Sugar-sweetened beverages	20.0	0.0 (reference)	-3.2 (-9.0, +2.7)	-1.6 (-6.2, +3.0)	-5.8 (-10.5, -1.1)	-5.3 (-10.4, -0.2)
Fruit juice		+3.2 (-2.7, +9.0)	0.0 (reference)	+1.6 (-2.1, +5.3)	-2.6 (-6.3, +1.1)	-2.2 (-6.2, +1.9)
Milk		+1.6 (-3.0, +6.2)	-1.6 (-5.3, +2.1)	0.0 (reference)	-4.2 (-6.6, -1.9)	-3.8 (-6.8, -0.8)
Coffee		+5.8 (+1.1, +10.5)	+2.6 (-1.1, +6.3)	+4.2 (+1.9, +6.6)	0.0 (reference)	+0.5 (-2.1, +3.1)
Tea		+5.3 (+0.2, +10.4)	+2.2 (-1.9, +6.2)	+3.8 (+0.8, +6.8)	-0.5 (-3.1, +2.1)	0.0 (reference)
Follow-up for 10 years						
Sugar-sweetened beverages	39.5	0.0 (reference)	-6.2 (-17.7, +5.2)	-3.1 (-12.0, +5.9)	-11.4 (-20.5, -2.2)	-10.4 (-20.4, -0.5)
Fruit juice		+6.2 (-5.2, +17.7)	0.0 (reference)	+3.1 (-4.1, +10.4)	-5.1 (-12.4, +2.1)	-4.2 (-12.2, +3.7)
Milk		+3.1 (-5.9, +12.0)	-3.1 (-10.4, +4.1)	0.0 (reference)	-8.3 (-12.9, -3.6)	-7.4 (-13.2, -1.5)
Coffee		+11.4 (+2.2, +20.5)	+5.1 (-2.1, 12.4)	+8.3 (+3.6, +12.9)	0.0 (reference)	+0.9 (-4.2, +6.0)
Tea		+10.4 (+0.5, +20.4)	+4.2 (-3.7, +12.2)	+7.4 (+1.5, +13.2)	-0.9 (-6.0, +4.2)	0.0 (reference)
Follow-up for 20 years						
Sugar-sweetened beverages	77.5	0.0 (reference)	-11.9 (-33.8, +10.0)	-5.9 (-22.9, +11.2)	-21.7 (-39.2, -4.2)	-20.0 (-39.1, -1.0)
Fruit juice		+11.9 (-10.0, +33.8)	0.0 (reference)	+6.0 (-7.8, +19.9)	-9.8 (-23.7, +4.1)	-8.1 (-23.3, +7.2)
Milk		+5.9 (-11.2, +22.9)	-6.0 (-19.9, +7.8)	0.0 (reference)	-15.9 (-24.7, -7.0)	-14.1 (-25.4, -2.9)
Coffee		+21.7 (+4.2, +39.2)	+9.8 (-4.1, +23.7)	+15.9 (+7.0, +24.7)	0.0 (reference)	+1.7 (-8.0, +11.4)
Tea		+20 (+1, +39.1)	+8.1 (-7.2, +23.3)	+14.1 (+2.9, +25.4)	-1.7 (-11.4, +8)	0.0 (reference)

[1] Crude incidence is presented as per 1,000 adults × 10 years (10,000 person-years), calculated as average across eight countries in EPIC-InterAct (774 cases / 192,287 person-years in subcohort). Estimates were obtained among those consuming ≥250 g/day of each beverage, leading to a specific incident rate and public health consideration.

[2] Values represent incidence rate over 1000 adults × 10 years for effects of substituting one of alternative beverages for one beverage. The difference between two rates (incident rate difference) was calculated as an multivariable-adjusted estimate of substitution effect under assumption of causality and a constant population-average incidence over time. Prentice-weighted Cox proportional hazard regression was modelled for each country. Country-specific estimates were pooled by multivariable random-effects meta-analysis. All analyses adjusted for recruitment centers, age, and sex. Further adjustment for potential confounders included education, marital status, hormone replacement therapy, menopausal status, history of oral contraceptive use, hypertension, dyslipidemia, family history of diabetes, prevalent diseases (coronary heart disease and stroke), smoking, physical activity, alcohol consumption, dietary supplement use, and dietary consumption (total energy intake, vegetables, fruits, nuts, cheese, yoghurt, red meats, processed meats, fish, confectionary, and cereals).. All beverages were mutually adjusted. 95% confidence interval (CI) was calculated by bootstrapping estimation.

Supplemental Table 7. Prospective associations of selected beverages with incidence of type 2 diabetes in sensitivity analysis varying groups of beverages: the European Prospective Investigation into Cancer and Nutrition – InterAct case-cohort analysis (*n*=27,662)[1]

Scenario	Each beverage evaluated separately[3]	Hazard ratio (HR) (95% confidence intervals, CI) per 250 g/day [2] SSB	Fruit juice	Milk	Coffee	Tea
Adjusted for potential confounders plus BMI and waist circumference (*n*=27,662)		1.18 (1.08, 1.28)	1.06 (0.96, 1.17)	1.10 (1.02, 1.19)	0.91 (0.89, 0.94)	0.93 (0.87, 0.98)
Substituted for SSB per 250 g/day		1.00 (reference)	0.89 (0.74, 1.07)	0.91 (0.82, 1.02)	0.79 (0.72, 0.88)	0.78 (0.72, 0.85)
Sweetened milk beverages,						
Not included as SSB		1.16 (1.07, 1.27)	1.05 (0.95, 1.17)	1.10 (1.02, 1.19)	0.91 (0.88, 0.94)	0.93 (0.87, 0.98)
Included as milk, not SSB		1.17 (1.07, 1.27)	1.06 (0.95, 1.17)	1.11 (1.03, 1.20)	0.91 (0.89, 0.94)	0.93 (0.87, 0.98)
Evaluated separately (*n*=15,358) *	2.56 (1.04, 6.29)	1.24 (1.10, 1.41)	0.95 (0.88, 1.01)	1.09 (0.97, 1.22)	0.89 (0.86, 0.92)	0.97 (0.91, 1.04)
Substituted for SSB per 250 g/day	1.81 (0.70, 4.68)	1.0 (reference)	0.76 (0.66, 0.86)	0.83 (0.69, 1.00)	0.72 (0.65, 0.81)	0.81 (0.72, 0.91)
Decaffeinated coffee						
Not included as coffee		1.18 (1.08, 1.28)	1.06 (0.96, 1.17)	1.10 (1.02, 1.19)	0.92 (0.88, 0.96)	0.93 (0.88, 0.98)
Evaluated separately (*n*=19,943) *	0.92 (0.79, 1.06)	1.21 (1.12, 1.31)	1.04 (0.91, 1.18)	1.09 (0.97, 1.22)	0.90 (0.82, 0.99)	0.91 (0.83, 1.00)
Substituted for SSB per 250 g/day	0.77 (0.70, 0.85)	1.0 (reference)	0.87 (0.71, 1.06)	0.89 (0.79, 1.00)	0.76 (0.65, 0.89)	0.77 (0.69, 0.86)
Vegetable juice						
Not included as fruit juice		1.18 (1.08, 1.28)	1.05 (0.94, 1.17)	1.10 (1.02, 1.19)	0.91 (0.89, 0.94)	0.93 (0.87, 0.98)
Evaluated separately (*n*=15,756) *	98 (<0.1, >200)	1.17 (1.09, 1.26)	0.85 (0.55, 1.32)	1.02 (0.93, 1.12)	0.90 (0.85, 0.96)	0.94 (0.85, 1.04)
Substituted for SSB per 250 g/day	75 (<0.1, >200)	1.0 (reference)	0.74 (0.52, 1.06)	0.87 (0.76, 1.00)	0.78 (0.72, 0.84)	0.83 (0.74, 0.94)
Artificially sweetened beverages						
Included as SSB (as total soft drinks)		1.11 (1.01, 1.22)	1.05 (0.95, 1.17)	1.10 (1.02, 1.18)	0.91 (0.88, 0.94)	0.93 (0.88, 0.98)
Evaluated separately (*n*=22,912) *	1.01 (0.85, 1.20)	1.20 (1.05, 1.37)	1.05 (0.94, 1.18)	1.11 (1.01, 1.21)	0.91 (0.87, 0.94)	0.96 (0.89, 1.04)
Substituted for SSB per 250 g/day	0.78 (0.74, 0.83)	1.0 (reference)	0.87 (0.70, 1.08)	0.86 (0.68, 1.09)	0.88 (0.75, 1.02)	0.76 (0.65, 0.88)
Water						
Evaluated separately (*n*=16,157) *	1.02 (1.00, 1.04)	1.17 (1.02, 1.35)	1.00 (0.89, 1.11)	1.11 (1.03, 1.19)	0.90 (0.87, 0.94)	0.95 (0.90, 1.00)
Substituted for SSB per 250 g/day	0.87 (0.76, 1.01)	1.0 (reference)	0.82 (0.62, 1.08)	0.94 (0.81, 1.08)	0.76 (0.65, 0.90)	0.79 (0.73, 0.85)

[1] Subtypes of some beverages were assessed only in a subset of cohorts; n of adults providing each information was presented; otherwise n=27,662. Each of the beverages assessed in a subset could be treated differently, as presented. The different options were tested separately. For substitution between beverages, that for SSB is presented; other results were not materially different from the main results (Table 2).

[2] Prentice-weighted Cox regression models were fit separately to data from each country. Country-specific estimates were pooled by random-effects meta-analysis. All beverages in each model were mutually adjusted. The first right column indicates estimates for either milk beverages, decaffeinated coffee, vegetable juice, artificially sweetened beverages, plain water, or alcohol. Demographic covariates included recruitment centers, age, and sex. Further adjustment for potential confounders included education, marital status, hormone

replacement therapy, menopausal status, history of oral contraceptive use, hypertension, dyslipidemia, family history of diabetes, prevalent diseases (coronary heart disease and stroke), smoking, physical activity, alcohol consumption, dietary supplement use, and dietary consumption (total energy intake, vegetables, fruits, nuts, cheese, yoghurt, red meats, processed meats, fish, confectionary, and cereals), BMI, and waist circumference.

[3] Either milk beverages, decaffeinated coffee, vegetable juice, artificially sweetened beverages, or plain water.

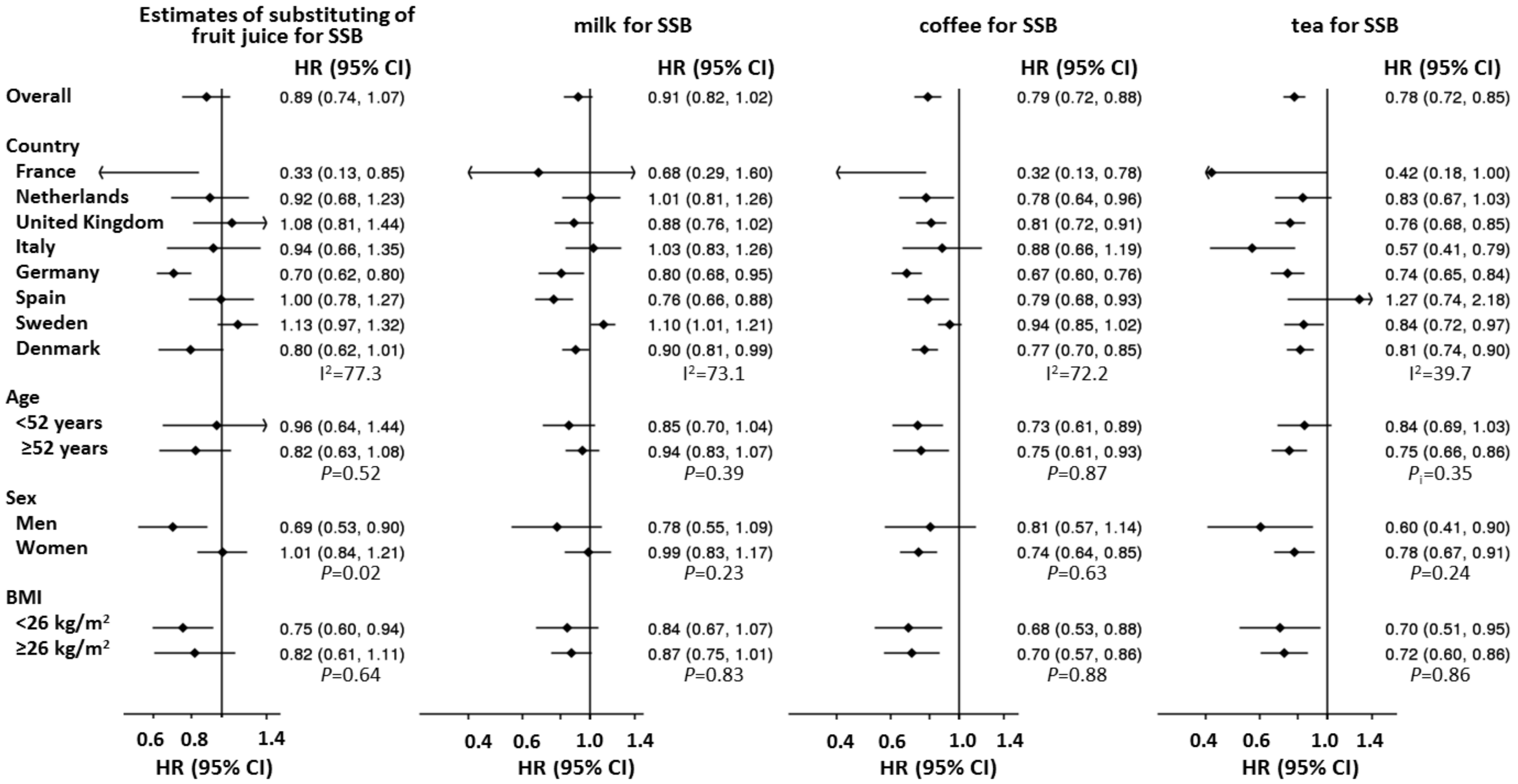

Supplemental Figure 3. Prospective results stratified by country, demographics, and body-mass index, representing potential substitution effects replacing sugar-sweetened beverages with consumption of alternative beverages (fruit juice, milk, coffee, tea) on incident type 2 diabetes: the European Prospective Investigation into Cancer and Nutrition – InterAct case-cohort analysis (*n*=27,662). Hazard ratios (HRs) and corresponding 95% CI were from pooled analysis (overall) or stratified analysis as indicated. Heterogeneity between countries was measured by I^2. There was no significant evidence for heterogeneity due to age, sex, body-mass index, smoking status, quality of dietary reporting, or absolute incidence (*P*>0.05 in meta-regression). For the other pre-specified factors, *P* for interaction are presented. Strata by age and by BMI were split by each median in subcohort.

Supplemental Text

Serum samples were stored from collection at up to −196°C in liquid nitrogen at the coordinating centre at the International Agency for Research into Cancer (IARC) in Lyon, France, or in liquid nitrogen in local biorepositories. As the exception, Umeå processed plasma samples and used −80°C freezers. All samples were shipped to the central holding bay in Cambridge, United Kingdom, where they were stored at −80°C until dispatch for genotyping or biomarker measurement. All available samples were then sent to the laboratory of the Stichting ingenhousz Laboratory, Etten-Leur, Netherlands, by 2010 July. All measurements were performed there using blood samples stored at -196°C (or -150°C in Denmark) from all InterAct cases (*n* = 12,403) and the random subcohort (*n* = 16,835).

In this study, we analyzed triglycerides, high-density lipoprotein cholesterol (HDL-C), high-sensitivity C-reactive protein (hsCRP) and hemoglobin A1c (HbA1c). Triglycerides and HDL-C were assayed with Cobas® assay adopting an enzymatic colorimetric test using Roche Hitachi Modular P analyzer (Roche Diagnostics International Ltd., Risch-Rotkreuz, Switzerland). The same analyzer was used for hsCRP for which particle-enhanced immunoturbidimetric assay was used. HbA1c was assayed with ion exchange high-performance liquid chromatography of Tosoh (HLC-723G8) (Tosoh Europe N.V., Tessenderlo, Belgium).

Supplemental References

1. The InterAct Consortium. Design and cohort description of the InterAct Project: an examination of the interaction of genetic and lifestyle factors on the incidence of type 2 diabetes in the EPIC Study. Diabetologia. 2011;54(9):2272–82. doi:10.1007/s00125-011-2182-9
2. Riboli E, Hunt KJ, Slimani N, Ferrari P, Norat T, Fahey M, Charrondière UR, Hémon B, Casagrande C, Vignat J, et al. European Prospective Investigation into Cancer and Nutrition (EPIC): study populations and data collection. Public Health Nutr. 2002;5(6b):1113–24. doi:doi:10.1079/PHN2002394
3. Slimani N, Deharveng G, Unwin I, Southgate DAT, Vignat J, Skeie G, Salvini S, Parpinel M, Møller A, Ireland J, et al. The EPIC nutrient database project (ENDB): a first attempt to standardize nutrient databases across the 10 European countries participating in the EPIC study. Eur J Clin Nutr. 2007;61(9):1037–56. doi:10.1038/sj.ejcn.1602679
